# Official Report on the Medical Topography and Climate of Calcutta, with Brief Notices of Its Present Disease Endemic and Epidemic

**Published:** 1841-01-01

**Authors:** 


### Official Report on the Medical Topography and Climate of Calcutta, with brief Notices of its prevalent Diseases, Endemic and Epidemic. By *James Ranald Martin*, Esq. Presidency Surgeon, and Surgeon to the Native Hospital. Printed by order of Government. 4to. Calcutta, 1839.

The "Notes" on the Medical Topography of Calcutta were noticed in a former Number, and Mr. Martin, in his preface, states that, to this favourable mention, and that of Sir James Macgrigor, is due the present enlarged Report, containing more than a third of matter entirely new.

From the preface also we learn, that to its author is due the comprehensive plan of calling on the medical services of all India for reports on the medical topography and climate of all the more important localities, and indeed of the country generally. This plan of Mr. Martin's, he states, "was carried into effect in 1835, through the direct act of the government, the medical board's opinions having afforded it but a very equivocal support."

Again, in 1838, it appears that Mr. Martin proposed to the same board to remodel the existing forms of hospital reports, which are now very defective, with a view to elucidate by medical statistics the important subjects of tropical climate, its diseases, and the results of treatment. "My plan," says Mr. Martin, "I regret to say, did not obtain the sanction of medical authority; and so this important matter remains, for the present at least, in abeyance."

The preface then concludes:—"There are two points which I have endeavoured to keep continually in mind—namely, the investigation of climate in its most extended sense, and secondly, its influence on military health; for it should never be forgotten here, however we may be circumstanced, that it is mainly for the care of the troops that medical officers are sent to India."

In a postscript we learn that the municipal committee, composed of the Honorable the Judges of the Supreme Court of Calcutta, the Lord Bishop of the Diocese, and several gentlemen, European and native, originated with Mr. Martin, and that he was a member of it. The report of this committee drawn up with singular ability by the chairman, the Honorable Sir John Peter Grant, and comprising 245 pages, is in our possession.

A glance at the improvements suggested by this committee will shew the extent of its labours, and the importance to the capital of British India of their being adopted—they are as follows:—

1st. "A new and complete system of drainage.

2nd. The more perfect ventilation of the city by the construction of new open streets and roads, and the removal of old buildings and walls.

3rd. The cleansing, clearing jungle, and levelling of ground in and about the city.

4th. Construction of large new tanks, and the supply of water for all purposes.

5th. Clearing and draining of the great salt water lake.

6th. Improved construction of the native habitations.

7th. Widening, paving and making streets and roads.

8th. Improvement of the public markets, &c. &c.

9th. Better regulation of the police, general and medical.

10th. Removal and regulation of burying grounds.
11th. The establishment of a great central hospital for the reception and cure of natives suffering from fever and other diseases incident to the climate, and the establishment also of dispensaries dependent on it.
12th. The various rents, taxes, assessments and tolls.
13th. The lottery for the improvement of Calcutta.
14th. The conservancy in all its parts.
15th. Suggestions for new modelling the police.
16th. Plan of taxation for perpetuating and carrying on the improvements of the city and its suburbs."

"The mere perusal," says Mr. Martin, "of the above abstract, will afford a sufficient coup d'œil of the actual condition and wants of the city." "That there are causes constantly in operation here, *and which may be easily removed*, that tend to produce great public unhappiness, by abridging both the usefulness and the term of life, any one may see; that it is the duty of the government—the only moving power in India—to obviate all these causes, no one can reasonably doubt."

Passing over the details of the medical topography of Calcutta, we come to the following observations, prefatory to a description of the physical climate of Bengal.

"Whover considers climate, with reference to its vast importance to human welfare, must feel some degree of disappointment, if not astonishment, at the meagreness in which the advanced state of knowledge in the nineteenth century has yet left this most interesting branch of inquiry. One philosopher will view climate as any spacc distant from the equator and poles; another, as nothing more than a well arranged table of the winds, of the thermometric, barometric and hygrometric degrees; a third, as having reference solely to elevation above the mean level of the earth's surface; a fourth, as consisting only of the internal heat of the globe; while a fifth, supposed to be better informed than all the rest, pronounces climate to be influenced only by latitude and local elevation, and allows it to be but slightly affected by any other causes. We may, then, with some shew of reason exclaim with Dr. A. T. Thomson, what is climate?"

Mr. Martin considers that, in this inquiry, "much that is important may be done by a careful observation and comparison of facts, made at different times and places; for it is by such means that a science like that of climate can alone be perfected." * * * "The value of all scientific facts depends in a great measure on being comparable, and this in an especial manner applies to inquiries relating to climate and medical statistics. I am satisfied that, in a professional sense, it is impossible to take too extended a view of climate, and that he who succeeds best must follow the indication of Cabanis—l'ensemble de toutes les circonstances naturelles et physiques, au milieu desquelles nous vivons dans chaque lieu; for this much is certain, that the framers of elaborate tables of the winds and the degrees of the thermometer have as yet done little either to inform our minds or guide our inquiries."

After quoting the arrangement of Malte-Brun, and adding to it, the author states that, "by tracing these causes, and by uniting and arranging under general points of view, the results of particular local observations, we shall arrive at an approach to climatology, in some measure corresponding to the present state of the other sciences."

The next article is on the "Influence of the Hindu Superstition and

Morals on Health." It begins as follows :—" We have not to travel to the banks of the Ganges to learn that the uninstructed man cannot even read the true character of the Deity ; that the works of Nature address their eloquent language to him in vain, that the destiny of man is obscurely beheld by him, and that it is only as he advances in knowledge that he becomes *capable* of receiving and comprehending divine truths, abandons ceremonies stained with blood, and tries to imitate the goodness which he then alone discerns in action all around him." The moral effects of caste are described by the author as follows :—" If we take a general survey of the institution of caste amongst the Hindus (and without doing so we can know nothing of the natives) we are inevitably led to the conclusion that, it wars with every passion of the human mind, good as well as evil, and that, being prejudicial to public happiness, it is eminently injurious to public health. In it we find none of the purifying influences of the Eleusinian rites of the Greeks, whereby both Isocrates, and Cicero in later times, considered that their morals and civilization were so much advanced." * * *

" On the contrary, in the Hindu act of devotion there is not a vestige of reference to the divine attributes, nor to moral duty. The Hindu rehearses in his mind the form of the God, his colour, the number of his heads, eyes, hands, &c. and nothing more; and he who preserves his caste need not disturb his mind on any other subject connected with moral or religious obligation. If there is any part of his conduct with which his religious ideas have no concern, it is his moral character."

The physical ills of caste are not less numerous or important. They comprise the early marriages, which Mr. Martin states to be " one of the most pervading injuries inflicted by caste on public health ;" polygamy also " that source of a thousand evils ; festivals of frequent recurrence, leading to every species of vice, exposing thousands to all the inclemencies of season, producing every variety of disease and misery, mental as well as bodily."

On diet Mr. Martin observes—" It has always appeared to me a great mistake to view the diet of the Bengalee *as prescribed by climate :* on the contrary, I believe it to be far below the standard required for his support under all the changes of his seasons: in the hot weather and rains it is insufficient to supply the great waste, and in the cold season its poverty is alike injurious to health."

" Whether the founders of the Hindu faith were aware of it or not is immaterial to the present inquiry; but there can be no question that by depressing all the physical energies, through a diet purely vegetable, they fastened with a stronger hand the moral bonds of Brahminical domination on the people."

The remarkable fact is there stated that, owing to the better food and clothing of the Mahomedan portion of the inhabitants of Calcutta, but one in $38\frac{1}{2}$ die annually, whereas of the Hindus, the annual mortality is one in $17\frac{1}{3}$.

Prefatory to an account of the institutions for the education of the natives, we have the following observations ;—

" Two circumstances appear calculated more than all others to keep the people in their present state of moral degradation ; the first and greatest is the caste ; the second is the want of any national plan of education."

The extinguishing influence of caste has been elsewhere spoken of; and here it only remains to remark briefly on the other great evil—the absence of education."

"In most European communities, education is a national affair—a part of the political system—and has for ages been subordinate, in the best regulated of them, to what is termed the law of society, and to that assemblage of opinions, customs, and habits which is not inappropriately called, by some writers, the positive morality of society, or the law of opinion. Now, here the people have never had any of these; and so long as they and education are wanting to the society of India, it cannot possess a shade of sovereign or independent quality, but must remain in its present anarchy. The people must look upon us as strangers, and we must look on them with the reserve of conquerors."

On the prevention of disease, Mr. Martin observes:—

"The admirable rules prescribed by Dr. James Johnson regarding dress, food, drink, exercise, sleep, bathing, &c. &c. and the regulation of the passions, are well known; but perhaps better known than regarded: they are like the vital points in religion and morals—all men agree in them, yet how easily are they forgotten! In order to think seriously on health, most men require to suffer from disease: the lessons derived from such experience are longest remembered."

"There is one circumstance which ought to be impressed every where on the public, and it is, that however useful medicine may be in moderate and judicious doses, under occasional circumstances of change of season, or during certain epidemics; it is yet more on the proper selection of localities, the avoidance of day and night exposure, and care in diet, exercise, clothing, &c., that disease is to be prevented, and not by a system of self-quackery, with calomel and other mercurial preparations, such as many persons pursue in this country to their great injury, for the removal of what they call 'biliousness.'

Many is the strong habit I have seen impaired by this senseless custom: and I have known several lives lost, and others put in jeopardy, by the use of saline purgatives during seasons of cholera."

"Another extensive source of disordered health I must here mention, as it has come frequently under my notice; I mean the long-continued use of aperient medicines containing the mercurial preparations. It is common for patients to obtain from their physicians aperient pills, for instance, containing some portion of calomel, or blue-pill. This may have been given with a particular view, or for an especial occasion only; but it often happens that the patient continues for months, and even for years, that which was intended to be used but for days, or weeks. The results are very lamentable—I have seen persons in a state of nervous irritability, bordering upon insanity, from this cause, with a subacute inflammation of the mucous digestive surface, and chronic ptyalism —all resulting from the long-continued and frequently unconscious use of mercury.

One field officer used blue-pill and colocynth for two years and a half; and an American gentleman took the same preparation with ipecacuanha, during a voyage from a sister presidency to America, and back to Calcutta. It is needless to detail how ruined were the healths of both."

On the physical management of European children in Calcutta, Mr. Martin observes—

"The diseases of childhood run their courses very mildly in Bengal, and, upon the whole, it cannot be said that, under proper management, the climate of Calcutta is unfavourable to infant health up to five or six years of age, when, however, the offspring of Europeans generally begin to shew the necessity for the change of climate, by out-growing their strength. This portion of medical statistics, however, is quite as unsatisfactory as all that relates to the subject in India; but I believe the results of a close observation would afford corresponding facts to those obtained in France and England, viz. that the greater mortality exists under the extremes of temperature—the very colder, and the hotter months."

The remarkable fact is then stated, that "amongst the better classes of European adults in Calcutta the ratio of mortality is greater than that of infants in the proportion of five to one, while of the poor degenerate Portuguese, that of children exceeds adults in the ratio of four to one."

Fort William is stated, on the authority of Dr. Burke, to be one of the worst, if not the very worst, of the military stations in India for the soldiers' children, the annual mortality being 16·29 per cent.

Lind speaks of "child-bearing as peculiarly fatal" in his time, but it appears now to be quite the contrary; for, during sixteen years that Mr. Martin was familiar with the state of health of the better classes of Europeans, but one death occurred connected with parturition. The mortality spoken of by Lind, Mr. Martin ascribes to the management having, in the olden time, been left entirely to native nurses, whose ignorance is said to be dreadful in its consequences to all who venture to trust them.

On the subject of rearing children entirely in Bengal, Mr. Martin observes:—

"That it is impracticable on the ground of experience, for that after much care the third generation from unmixed European stock is nowhere to be found; so much for the question of European colonization, on which a great deal has been said and written here, without ever reflecting that Nature had already set her ban upon it."

We come next to a chapter "On the Mortality of the Native and Foreign Races"—a task of great labour and difficulty, owing to the neglect of medical statistics by the medical authorities in Bengal.

This subject Mr. Martin has repeatedly urged "in what he thought the right quarter, and in the most emphatic manner; but though his proposition met with no very flattering reception, he has yet the satisfaction to know that he has produced some action, tardy perhaps, yet such as will lead to some ultimate improvement."

"We are in India continually kept in mind of that law of our nature by which the activity of men decreases in the ratio of their senility, causing that inertness and disinclination from undertaking any thing, however excellent, of which they cannot be expected to see the end."

Prefatory to elaborate tables, and the comments upon them, Mr. Martin observes that—

"In any inquiry as to the duration of life, and the causes of mortality amongst the natives of Bengal, we must consider, not only that general climate and temperature have great influence upon the longevity of different races, by accelerating or retarding the development of the human system, but that along with the worst climates, all the institutions and habits of the Bengalees tend powerfully to abbreviate the term of life: their premature decay is in perfect accordance with their early and forced development."

"The law of correspondence of the period of puberty with the whole term of life is subject to few exceptions, and has been well expressed by Lord Bacon in his Historia Vitæ et Mortis, by 'Natures's finishing her periods in larger circles.'"

By a table, at page 168, we find the annual deaths in Calcutta as follows:—

| | |
|---|---|
| English and Eurasians .. .. .. .. | 1 in 28 |
| Portuguese .. .. .. .. .. | 1 in 8 |
| Mahomedans, Moguls and Arabs .. .. | 1 in 36 |
| Hindus of various races .. .. .. .. | 1 in 16 |
| Armenians .. .. .. .. .. | 1 in 25 |
| Native Christians .. .. .. .. | 1 in 14 |

After a commentary on the above table, Mr. Martin observes that, "so injurious is the climate of Bengal Proper to the Hindu soldiery of the army, in comparison with their own climate of the upper provinces, that, although only one-fourth of the troops are stationed in Bengal, the deaths of that fourth are more than a moiety of the whole mortality."

"That it was not less fatal to their Mahomedan predecessors of the Mogul dynasty is evident from the following translation of Gladwin, from the Persian:—In former reigns the climate of Bengal, on account of the inclemency of the air and water, was deemed inimical to the constitution of Moghuls and other foreigners, and only those officers who laboured under the royal displeasure were stationed there; and this fertile soil, which enjoys a perpetual Spring, was considered a strong prison, a land of spectres, the seat of disease, and the mansion of death."

The average mortality of the European soldiery in Fort William is stated in a table, for 20 years, at $76\frac{3}{4}$ per thousand of strength; but Mr. Martin thinks that, including invalids who die within a year of their departure from India, and the correction of other errors, the actual mortality will approach 90 per thousand.

A table is then given to shew that in India, as elsewhere, age materially influences mortality, the annual ratio per thousand of colonels being 59—4, while that of ensigns is but 23—4.

A series of tables is then given to shew the influence of season on the mortality of both natives and Europeans. They all agree in proving that the most fatal period of the year is from September to January inclusive; November being the month in which the greatest number of deaths occurred from ordinary endemic disease, while May is found to be "the worst of the cholera months."

The next article is on the epidemic disease of Calcutta.

"I know no branch of medical inquiry more interesting than the history of epidemics, or one that would prove more useful to practitioners in whatever climate. How true it was, as declared by Sydenham (and it unhappily continues so to this day) that no one has treated this great question 'in proportion to the dignity of the subject."

"A history of our local epidemics would be of great value, as enabling us to trace their connexion with changes of climate and condition of the surrounding localities, or with social conditions of the people, both European and Native; and had such an history existed, it would have helped to an earlier establishment of general principles, and a rational plan of treating our fevers especially; for, though all the epidemics within my personal recollection (and there is scarcely a year we have not one in some form) differ essentially from the ordinary endemics of the country, still, there will be found in most of them, so much of the savour of the soil, if I may be allowed the expression, as to render a knowledge of their history and treatment an object of no mean importance."

Mr. Martin then goes on to state that "endemics are very often the parent stock upon which epidemics are engrafted;" but that our means of prevention apply equally to both, inasmuch as each "are found to *fasten with peculiar severity, and remain longest in such localities as are neglected.*"

### Cholera.

"It is impossible to say any thing satisfactory on a disease, which would seem every year to become a greater source of difficulty to medicine. In the stage of collapse it has too often proved to physicians what traumatic tetanus is to surgeons, a disease, according to Hennen, which, once fully formed, tended more to shew him what he could not trust to, than what he could place the smallest reliance on. My notices of cholera, then, will comprise merely such desultory observations respecting the local history of the disease and its treatment, as my personal experience in various parts of India and Ava enables me to offer; for, as to its remote cause, little or nothing is known: like the pestilences of the fifth century before the Christian æra, it has in a manner travelled up and down over the habitable world, returning often to the same place after a certain interval; pausing sometimes in its fury and appearing to sleep, but again breaking out on some point or other within its range, till, at the end of its appointed period, it disappears—I fear we cannot yet say—altogether. The first impression of the morbific causes would appear to be made on the system of organic nerves and their functions; for almost immediately we have the vital actions of circulation, respiration, the generation of heat, and secretion, conspicuously disordered, and that probably through some unknown changes in the electric condition of the atmosphere."

"When I first entered on military practice in 1818, the disease had some marked points of difference in the symptoms and in the means used to combat them, from those of more recent visitations of the epidemic. Formerly, simple venous congestion of the most aggravated nature seemed to form the most essential feature in the disease; and the spasm, which was of a chlonic character, could be referred to the oppression of the nervous energy following on concentrated cerebral and spinal congestion. It is on the supposition of the condition stated forming the leading feature of the disease in former times, and of the organic nervous function being less involved, that I would account for the efficacy of blood-letting then; for latterly, there seems a depression of the vital actions, which has greatly embarrassed our treatment, and deprived us altogether of the former great resource, blood-letting."

To illustrate the difference here spoken of, Mr. Martin states the cases of two men whom he treated very early in the history of the epidemic in Bengal, and in his own practice;—one was that of a soldier of the 59th Regiment, in whom the spasm was so severe that it required four men to hold him down. "He was bled profusely, and in a few hours he recovered." The other case took place some years later in the Governor-general's body-guard of cavalry.

"I found that during the night he had been drained of all the fluid portion of his blood; his appearance was surprisingly altered. The respiration was oppressed, the countenance sunk and livid, the circulation flagging in the extremities. I opened a vein in each arm, but it was long ere I could obtain any thing but a trickling of dark treacly matter; at length the blood flowed, and by degrees its darkness was exchanged for the hue of nature. The man (an European), though not robust, was bled largely, and he, whom but a moment before I thought a dying man, stood up and exclaimed, '*Sir, you have made a new man of me.*' How surprising to find that a few years more, and the same treatment would, within the same time, have proved as certainly fatal. To appearance, we have the same symptoms, in the same order; but these signs are fallacious; and the treatment which proved most successful if used early in the disease, during 1818—22, no man will now dare to put in practice :—why is this? Is it that in the more recent visitations of the epidemic, we have, from its very onset, a greater depression of the organic nervous energy than was observable during its earlier history? I think so, and that such depression constitutes the chief, if not the entire difference now-a-days."

"Since the first outbreak of the epidemic in Bengal, it has spread westward, and over the nations of Europe and of America; yet, on a careful review of its history in all these countries, it does not appear that any additions whatever have been made, either to the Indian pathology or treatment of cholera. I do not make this assertion in disparagement; far from it, but to shew the great difficulties of these questions."

"Of the pathological state which actually constitutes the disease, we shall probably never know much. There is, in rapidly fatal cases, a great exhaustion of the power of generating heat; the air expired from the lungs becomes progressively colder; and so do all parts of the body, until they are merged in that of death; but these and the other destroying states already mentioned leave no traces behind."

"In protracted cases again, congestions, and even inflammations of various organs are frequently discernible after death; but these form only consecutive, or superadded events in the morbid chain, like the final oppression of the cerebral system of nerves, which occasionally become obtunded through the stagnation of the circulation, and consequent want of red blood. In the last stage of acute seizures, however, it is surprising how some retain their mental integrity to within a few minutes of dissolution, while others are for hours narcotized by the disease."

"Here, as in all epidemics, it may be laid down as certain, that whatever tends to disturb the balance of health, may lead to an attack of the prevailing disease. Hence, it is to the observance of preventive rules that communities will always owe most."

"The avoidance of day and night exposure, and, in short, all those rules applicable to ordinary prevention should here be rigorously attended to. The diet at such seasons should be nutritious, dry, and moderately stimulant, and all food should be easy of digestion. I have known several instances wherein the use of the shell and sable fishes of this river have led to an attack of cholera, apparently through their difficulty of being well digested. Cold ascescent fruits and vegetables ought likewise to be avoided. An empty state of the vessels, hunger, thirst, fatigue, and debility, give rise to activity in the process of absorption, and this would appear to explain how persons enfeebled by debauchery and loss of natural rest are more liable to be affected by diseases, whether epidemic or contagious. It is thus also that fear would seem to act. Another caution applies to the use of purgatives, and especielly those of a cold saline nature: many cases have occurred within my recollection of the disease being thus insinuated and mistaken for the action of salts, until the speedy approach of the stage of collapse made the real nature of the case but too apparent. When purgatives must be used in such seasons, they should be of a warm aromatic nature, and it ought to be a fixed rule never to exhibit them over night, that being the ordinary time of invasion. It must not be forgotten too, that fevers, and bowel complaints especially, are very apt, under the epidemic influence to merge in cholera."

TREATMENT.

" Details of medical management can have no place in a work of this description; but this much must be evident, viz. that in a disease, acute beyond all others, treatment, to be successful, ought to be applied at the onset; life may then be saved in a very large proportion; but, if unhappily the first few hours are lost, it too often follows that unavailing regrets take the place of hope, and that the best efforts of the physician are set at nought.

I have stated that the plan by copious bloodletting, followed by full doses of calomel and opium, was that found most successful on my arrival in the country, in resisting the onset of the disease; and when re-action took place, topical bleeding was used to relieve local affections, and repeated mercurial purgatives completed the cure."

" When, however, no such happy condition was present, but the opposite one of collapse of all the vital powers occurred, we were then, as now, very much wanting in an available resource, notwithstanding the free use of every mode of stimulation."

" It is very true that we occasionally see surprising instances of recovery from apparently hopeless sinking of the nervous and vascular energies; but candour will oblige all men to avow that, though such cases are saved by the assiduous exhibition of stimuli, yet it is equally true that the majority of them die now, as they did in our earlier experience; and I repeat that our European and Transatlantic brethren have not helped us through any of our difficulties in the management of this stage of the disease."

" A full dose of calomel with opium will save life in a majority of cases if used early;—and this fact ought to prove a great encouragement to all who will think on the subject as it deserves.

It will be seen then, that excepting the blood-letting, which we have been obliged latterly to abandon as deadly, our practice remains much the same it was at the commencement of the disease in 1817:—that is, the stimulating plan of treatment, first adopted here, seems to have now become the general one in all countries."

" It appears that in the Calcutta general hospital, out of 803 Europeans, admitted in all stages of the disease, 372 died: at Madras 23 per cent. of the Europeans and 45 per cent. of the natives died; while in England, 38·5 per cent. died; in Prussia 58·6; in France 100,000 out of the entire population. March, April and May are the months in which the disease generally prevails, but May is much the most fatal.

In England half the deaths occurred in the first 24 hours, shewing the urgent necessity of early treatment in this disease; for, what is to be done must be on the instant. In the epidemic visitation of 1838 in Calcutta, the deaths that I witnessed took place from two to twelve hours from my first seeing the sufferers. Here there was time—every thing in cholera—irrevocably lost."

Passing over the history of such other epidemics as came under Mr. Martin's personal observation, we come to the endemic diseases of Calcutta, and first, of Intermittent Fever.

It appears that, owing to the " hitherto gradual and almost imperceptible improvements of the ill-chosen locality, intermittent fever has become a mild and infrequent form of disease in modern Calcutta, while in former times it constituted far the most severe and fatal class of fever; the cold stage, according to the older writers, lasting " *twelve hours*."

"The fevers here mentioned would seem to have possessed the malignant character of the *febres* intermittentes algidæ, described by Torti, in which the power of generating heat was so impaired that the patient died in the cold stage at the end of two or three accessions."

#### TREATMENT.

"With exception to the cases of some delicate females, I do not recollect any that resisted the ordinary management by general or local blood-letting, according to the severity of complication; purgatives and sudorifics, with quinine during the intermissions."

Mr. Martin's experience does not enable him to speak favorably of bleeding in the cold stage of intermittent. On the contrary he recommends that, "when general blood-letting is had recourse to in the treatment of intermittent fever, whether simple or complicated, it should, as in the case of all other fevers, be performed at the very onset of the stage of re-action."

"Practised at this period, it will lessen arterial action, relieve venous congestion, usher in the sweating stage, and thereby pave the way for quinine, purgatives and sudorifics, on which the prevention of recurrence must depend."

"In feeble habits, or with persons who have resided long in India, local depletion, by leeches, will answer every purpose."

### REMITTENT FEVER.

"In noticing the more ordinary fevers of Calcutta, whether endemic or epidemic, the first observations that force themselves are, their great differences as to intensity, in the present, as compared to former times; and secondly, the causes of this difference."

"The earliest account we have of the state of public health, and of the period of greatest mortality, in Calcutta, is that already quoted from Captain Hamilton, wherein he mentions 460 burials out of 1200 British inhabitants, from August till the ensuing January: this was between the years 1688-1723."

Notices are then given from Clark, Stavorinus, Ives, Bogue and others, of the fatal nature of the fevers of Calcutta in their days.

"That a scorbutic taint was universal in those times may, I believe, be admitted; and this circumstance will readily account for the general term 'putrid,' as applied by the older writers to the endemic fevers and dysenteries: this unfavorable complication will likewise go far to account for the mortality."

"Of just or useful comparison therefore, between the results of former and recent general hospital management, there can be little or none; but, I think, this much may be allowed in favor of the modern plan; that, in consequence of our greater freedom of depletory means, by blood-letting especially, we have fewer of the sequelæ of fevers, viz. enlarged liver and spleen, than existed formerly; for, in the military hospital at Madras, in 1782, we find by a monthly report of Mr. Paisley, the surgeon, that there were then in the house—

| | |
|---|---|
| Venereals | 50 |
| Quotidian remittents | 2 |
| Simple bilious fevers | 30 |
| Bilious fevers, with visceral obstructions | 15 |
| Simple fluxes | 20 |
| Liver fluxes, and fluxes from visceral obstructions | 98 |
| Chronic visceral obstructions from impaired habits | 69 |
| Total | 284 |

"Thus it appears, that out of 284 cases in hospital, 182 laboured under some form of visceral obstruction; that is organic disease of the liver or spleen, or both, in a more or less acute form, and in either case rendering death more or less remote, a necessary result.

It is here then, and not in the comparison of actual mortality within the wards, that our hospital management contrasts favorably with that of the olden times."

"If tropical fever and dysentery were always simple morbid actions, no doubt, as recommended by some, bleeding and purging might in general prove adequate to the cure; but unfortunately in both cases, we seldom find this unmixed condition to hold in actual practice in Bengal, where we have in our fevers continually to combat dangerous abdominal complications, with the addition, in the hot season, of the cerebro-spinal—all demanding a more or less complex and careful treatment—a speedy unlocking of all the secretions and excretions, which the most ample experience proves that bleeding and purging *alone* will not effect. Yet bleeding, here, as in dysentery, is the standard remedy, subject to age, constitution and length of residence in India. It precedes all other management in the order of time, and in point of importance. I believe this to be the general view taken of it by the practitioners of this city; and it is but common justice to say, that the value of this most powerful of all means was first emphatically urged on the Indian surgeon by Dr. James Johnson: it is to him we owe that blood-letting has become a systematic part of our treatment. Of the several valuable authors besides, who have since followed him, and helped to fix the professional attention, I need say nothing."

"Subject only to the limitations already stated, bleeding—early and copious bleeding, and practised at the very onset of the stage of re-action—is very generally necessary in the severer forms of Bengal remittent fever; then, full doses of calomel with sudorifics, short of producing salivation, with saline purgatives in the intervals. If the disease does not now yield, but on the contrary, if the paroxysms recur at shorter intervals, and with increased severity, leaving but imperfect remissions—then there is imminent danger, and inflammation or acute congestion in some important abdominal or other organ, may be more than suspected. For this, in addition to topical bleeding and cold to the head, when the seat of disease, mercury in small repeated doses, with antimonials, must be given so as mildly to affect the system: it is the only known means of saving the patient, by anticipating the destruction of some organ essential to life; it here becomes, in the apt words of Dr. Robert Jackson, a remedy of necessity."

"Where the remissions, on the other hand, are well marked, quinine should be given in full doses, without waiting for every thing. Some practitioners recommend that, before this drug is used, we obtain previously a clean tongue, natural secretions, and the absence of all heat of skin or local affection. I believe this to be a very dangerous practice: if we are to wait for every thing, we shall often wait too long, or till it is too late. I have always administered quinine in the more favourable cases now stated, in disregard of certain local abdominal complications, (those of the head should in general exclude it,) beleiving that if I arrest the paroxysm, I do greatly more towards the cure at large, than quinine can possibly do of harm to the local affection—the treatment of which by local depletion and counter-irritants is not interfered with by this means: again, all tenderness on pressure or local pain, does not, in the case here stated, necessarily constitute inflammation."

"Almost all our complications in the fevers of Bengal are abdominal, whether these be of an inflammatory nature, congestive, or of mere irritation; and this would seem to be the cause of the apparent prostration, with tendency to collapse, so common especially during the rainy season with us; for even within a few hours, as contrasted with similar affections of the head and chest, there exists here an oppression of the vital functions, alarming to the stranger physician."

"The prostration produced by a violent blow on the abdomen more nearly resembles the febrile collapse than any other morbid condition with which I am acquainted; it is probable that both depend on the disturbed function of the great sympathetic—the powerful though silent source of many symptoms known to us only by their effects."

"This tendency to sinking is the reason why our measures of cure must be so guarded as to the time of using them;—for there is no country in the fevers of which more regard must be paid to the stage of disease for applying remedies, especially blood-letting, than in those of Bengal; what was a saving means at the commencement of the paroxysm, is as surely destructive at the end of it."

"I cannot conclude these cursory remarks without adverting to the importance of the management of convalescence from fever—not the least serious of the duties imposed on the Indian physician. In all cases of recovery from fever, but especially in those wherein the complications have been severe, or where important organs have been affected in the course of the fever, or as a sequel to it, it is impossible to be too careful in the diet, and in attention to the nature and activity of the secretions;—and this vigilance must not be relaxed until perfect health is re-established. How often do we see patients who have been well enough treated during the acute disease, but on whom the neglect of this rule of practice entails enlargement of the liver or spleen, or other visceral engorgements, requiring a protracted sea voyage, or even a return to Europe at great inconvenience. This is a subject that should always be present to the mind of those who have the management of military hospitals wherein the perfect re-establishment of the soldier's health, before his return to barracks, should be a maxim never to be swerved from."

"This is not the place for reviewing medical books, or laying down general rules of practice; but it cannot be too much or too often impressed on the Indian surgeon, that it is on his careful attention to the phenomena of fever that nine-tenths of his usefulness depend. I have here attempted an outline of the treatment of the endemic fever of Calcutta, and of Bengal generally: it will be found to correspond in principle with that of the endemic fevers—the bilious remittents of the world—whether east or west:—they are all fevers of locality, and do not by any means differ so much as medical writers of partial views and partial experience would have us believe:—their supposed differences, or nosological divisions, are more frequently the work of man than of nature: they may, and do differ in degree of intensity; but their essential phenomena, and the organs affected in their progress, so as to endanger, or ultimately destroy life, are the same; and so likewise are the essential parts of their treatment."

### Dysentery.

"If dysentery were a disease of uniform character, and having an uniform *cause* and *seat*, then it might, perhaps, always be treated after an uniform plan; but a very slender experience of this disease, especially as it prevails within the tropics, or even within the British Islands, shews this not to be very generally the case; for although some portion of the larger bowel is universally implicated, yet, either from the first, or during the progress of the disease,—for we cannot often say which—the lesser bowel—the liver, the spleen, the pancreas, and mesentery, become also the frequent seats of morbid action, so as to modify the disease, and likewise its right treatment."

"In the dysentery of Ireland, Dr. O'Brien found 'the liver diseased in one-half of the dissections, the spleen in one-fourth, the small intestines in two-thirds, and the colon and rectum in all.' The pathology of our dysenteries, whether in Southern or Northern India, and as given by the best authors, sufficiently establishes that morbid action in this formidable disease is not confined here, any more than in Europe, to the course of the large intestine, but that all or most of the associated organs are found after death to be more or less deeply implicated, just in proportion to the extent and severity of the symptoms during life."

"It appears to me that to a want of just consideration of these inevitable pathological complications must we ascribe the system of exclusive treatment so much reprehended by the author quoted at the head of this article, and the successive abandonment also, by the surgeons of fleets and armies, of every exclusive plan hitherto proposed, almost as soon as it has been tried."

In this article, as in that on remittent fever, Mr. Martin gives a catalogue-raisonné of the treatment of these diseases as they prevail both in Europe and within the Tropics, by more than twenty of the best known authors, exhibiting some considerable variety in practice it is certain, but within latter years coming to a close agreement on the general principles of management.

"It only remains to notice the prevailing treatment of the dysentery of Bengal, amongst the more experienced practitioners at the Presidency, and this I shall insert in the order of importance. Blood-letting, general and local, as first practically urged in the dysentery of India by Dr. James Johnson, takes the lead, and has done so for many years: it is the standard remedy; and I believe that when the subject comes early and freely under this treatment, and that the case is not complicated with hepatic congestion or other actual disease, little else than a few aperients and sudorifics will be required for the cure; but, as in most cases of this formidable disease as it appears within the tropics, the diseased state of the large intestines is essentially mixed up with general abdominal complications, other and important means follow the bleeding; and of the first are those which act powerfully on all the secreting organs, internal and external —such as calomel in full doses with antimony, or with ipecacuanha, followed by laxatives, sudorifics, warm baths, enemas and other minor adjuvantia. I believe this to be the general course here, and I have seldom seen calomel carried the length of salivation, neither do I consider this degree of effect necessary to the cure."

"The late Mr. Twining, in his clinical work, advocates the use of simple ipecacuanha powder, combined with the bitter extracts, which plan he described as very successful. I am not aware that this system has been followed by any of the other practitioners of the general hospital, where Mr. Twining officiated for several years; neither would it appear to be successfully imitated in the provinces."

"Dr. Macnab, in a very judicious and practical report on the dysentery of the native soldiery of Hindustan, when serving in Bengal, says, that 'blue pill with ipecacuanha and gentian proved a complete failure,' as has generally been the case in my trials of it. Indeed I much suspect that Mr. Twining overrated the value of this favorite remedy, and that he may also have miscalculated the anti-emetic properties of the gentian."

"Ipecacuanha has been a favorite remedy in the south of India for upwards of forty years past. Dr. Whitelaw Ainslie, after an experience of thirty years, and an extensive practice amongst all classes of Europeans, says of this drug, that it 'has no equal in simple dysentery, that is, dysentery not accompanied with hepatic derangement; it has the happiest effects.' This is an observation of great practical importance, and, I think, impresses a just discrimination in the use of this valuable remedy. In speaking of an experience now of twenty years, and an extensive range of observation of the disease as it occurs in hospitals and private practice in this city, also as it appeared amongst the troops serving in the unhealthy provinces of Orissa and Guudwauah, and in the army at Rangoon and Upper Ava, I should say, with Dr. Ainslie, that it is alone in *simple uncomplicated* dysentery that ipecacuanha shows its best effects administered as an *exclusive* remedy; that is, after bleeding and moderate purging."

"In the hepatic form of dysentery—no uncommon complication in Bengal, especially during the cold season—calomel is absolutely necessary to the cure. I lately treated for this form of the disease a gentleman who had suffered much from the Batavian fever contracted at the capture of Java: he was bled generally and by leeches, followed by purgatives and sudorifics; but no amendment took place, and nothing was voided but mucus and blood. Two full doses of calomel and antimony were then given, which produced copious biliary discharges and immediate relief: a few doses of blue-pill with ipecacuanha, and purgatives, concluded the treatment. There existed in this case no enlargement of the liver, nor uneasiness on pressure; but there was a total absence of biliary secretion; and until that was restored, the other treatment afforded no relief. Another case of very severe hepatic dysentery requiring measures of great activity, was marred in convalescence by soup taken contrary to my directions: the liver became painful as ever, and the dysentery returned, requiring a repetition of general and local blood-letting, mercury, &c., and that under circumstances of greatly reduced strength. I have seen many cases in which morbid action seemed co-existent in the liver and cæcum, and I would beg to call attention to the subject. I believe that cases of hepatic complication, treated without mercury, frequently terminate in inflammation and chronic abscess of this organ."

The last article but one is on the inflammation of the parenchymatous structure of the liver.

"This is the most dangerous disease I am acquainted with, because of its insidiousness, and the total absence of urgent symptoms: the process which leads to destruction is here silent and rapid. It has not been sufficiently dwelt on by writers on the diseases of our climate. It chiefly attacks the feeble of constitution, the lax of fibre and fair of complexion: it often terminates the career of the old Indian."

"Whether existing in the older resident in Bengal or the new comer, it is generally a disease of the cold season, and caused by night exposure;—in short, by any means that determine powerfully from the surface to the internal organs. I have seen cases where it was caused by the chilling thorough draughts of our northern entrances to the Calcutta houses, after leaving a crowded room; and others, where it was occasioned by exposure before day-light for the purpose of hunting."

"The disease is sometimes preceded by a perceptible falling off in the general health, such as, some degree of emaciation, dry cough and embarrassed respiration, loss of appetite, and sallowness of complexion; but it more generally comes on in the midst of apparent health. There is a general feeling of abdominal uneasiness, but more particularly of the epigastric region and that of the liver, with some degree of fever, preceded by slight rigor or ague; but all these are so trifling as too often to attract but little of the patient's attention. Perhaps he applies to his physician on account of slight *diarrhœa*, supposed to be the result of error in diet: medicine affords some relief, and he proceeds in his ordinary occupation for days, and when the action is more chronic, for weeks, though under great depression of the mental and corporeal energies; till at length, his altered appearance, hacking cough, permanently dry skin, invincibly rough furred tongue, and morbid taste, attract some more serious notice on his own part, or that of his family. The real nature of the disease may still remain a secret to both patient and physician; and it may not be till a marked succession of rigors, or profuse and clammy perspiration announce in audible terms the formation of abscess, that either party becomes awake to the actual danger. A sense of uneasy feeling may or may not exist in the region of the liver, according as the disease is centred more or less deep in its substance, or in its upper convex surface ; when the former exists, the symptoms are more than usually obscure and insidious ; in the latter case, they are very acute."

"When on the other hand, the left lobe is the seat of morbid action, it is easy of detection to a physician, though not so to the patient.

"I should say that diarrhœa, followed by slight fever ; the peculiar state of the skin ; the tongue having the roughness of a coarse file ; with adherent coating, together with the local uneasiness already described ; cough, and high-coloured urine, ought immediately to warn the physician of the suppurative inflammation which leads to liver abscess. The diagnosis will receive material assistance from the external examination of the chest, especially when the upper convex surface of the liver is the seat of disease."

"Such are the symptoms and most uniform succession of events : they should always meet with the strictest attention, and the most prompt and decided treatment."

"I will give a case in illustration. Last cold season a medical friend called at my house, and just as he was quitting, he said, incidentally, that he had a 'pain *in his back,* like lumbago.' On examination, I found his liver seriously involved in disease, and that it had been so for three days, during which he had been going about as usual, living in his ordinary manner, and using the *cold* bath daily. All he had noticed was a slight shivering three nights previously, followed by feverishness and pain of the back ; but he considered his symptoms of so little moment that his mention of them was obtained only through interrogation."

"He was young and robust of habit, so that with the loss of about eighty ounces of blood within twenty-four hours, his symptoms yielded :—but I think he recovered with difficulty : a few hours more, and it would have been too late. The above is an extreme case of the kind, the inflammation having been of a very acute character ; but it is important, as shewing how very insidious are the symptoms, and how little they possess of the urgency to cause a salutary alarm in the patient's mind. It is always thus when the inflammation is centred in the parenchymatous structure of the liver ; and hence the absence of acute pain, and those urgent symptoms which characterize inflammatory states of the peritoneal covering of the gland, which always give ample warning."

"However long the disease may have existed, provided there be no symptoms of suppuration, general bleeding, copious in relation to age, health, and length of residence in India, must be instantly had recourse to, and the measure of depletion should be the sense of general relief, *with softening of the skin.* These are the only safe criterions of adequate loss of blood, and it should be continually held in recollection, that suppurative inflammation of the most deadly character is present, and that consequently there is no time to be lost. After the bleeding, calomel and antimony should be exhibited every four hours, with occasional smart purgatives in the intervals, until the system is brought mildly under the influence of mercury : leeches and blisters are of course useful, but the latter ought not to be applied till a powerful impression has been made on the disease. The diet, during the progress of treatment, and for a long time after, should be of the very sparest, such as thin sago or arrow-root. On a plan of cure such as this, I have seen cases of a very unpromising appearance end in health. Occasionally this has been finally effected by the steady use of the nitro-muriatic acid bath, persisted in for a month or six weeks. One error seems very general respecting this disease, namely, its supposed infrequency in Bengal ; but so much am I satisfied of the contrary that, taking the idiopathic cases of it, and those that form the complications with, or sequelæ to fevers and dysentery, the sum total would form a respectable item in our bills of mortality; indeed the statistical tables furnished in this work prove it."

The last article is on Neuralgia.

" Neuralgic affections, and especially the tic douloureux, prevail endemically in Bengal. We have here 'the true tic'—a terrible disease, that strikes with the violence and suddenness of lightning—the torture of which no language can describe.

" In such a disease no man can remain an unconcerned spectator; and with all our sympathies aroused, we have often to regret the inertness of remedies the most powerful in repute, such as tonics of all kinds, occasional purgatives, as general measures, and veratria, ice, and acupuncture, as local means."

" Dr. Elliotson assures us, that he has never seen one case of neuralgia referrible to disorder of the digestive organs, and it may be so in England; but I must say that, till my attention was drawn forcibly to this subject by Sir Charles Bell, I had no great reason to congratulate myself on my success against the tic of Bengal."

Mr. Martin, after quoting Sir Charles Bell, goes on to state his belief, that disorder of the digestive organs is, in Bengal at least, one of frequent influence.

" That such is a common source in the neuralgic affections of this climate, there can be no doubt; and acting on that impression, I have cured a great number of cases of the severest forms (tic especially) in which all other remedies, general and local, had failed. I allude to one case especially, that of a lady who had suffered for years, and used a variety of means under direction of the late Mr. Twining, who at last directed the removal of the teeth of the side affected; but which was not done. Her agony used to last for a fortnight together, so as nearly to deprive her of reason. The purgative plan pursued for two months removed the pain; and for three years she has had no return. I could add many similar cases to prove the general influence of intestinal irritation on the disease in question. No doubt cases may occacionally occur, where the plan here recommended, and all others, will fail of doing good; but such should not discourage us. I have succeeded by combining the purgative and tonic plans; indeed the first, pursued in the moderate way stated, interferes with no other measures of cure."

In the above analysis we have avoided all comments, not considering ourselves justified in questioning any of the opinions and practices of a gentleman, whose talents and opportunities are entitled to so much respect. The materials of this report are, indeed, so valuable, that the senior Editor has added them to a new edition of his work on Tropical Climates, now in the press. This fact speaks for itself. But we cannot dismiss the "REPORT," without alluding to a circumstance which must have been highly gratifying to its author, and which, indeed, is creditable to the medical profession in India, as it shews the estimation in which it is there held by the public at large.

In January 1840, Mr. Martin, after a long residence in India, determined to husband out what health was spared, by a return to his native climate, and he is now settled in this metropolis, where we are sure many of his old Bengal friends and patients will find him out. The following extract from the Calcutta Courier, of January 15, 1840, will shew the estimation in which Mr. Martin was held by the inhabitants of that great Presidency.

" We are informed that the friends and patients of J. R. Martin. Esq., Presidency Surgeon, have determined to present to him on the occasion of his approaching departure from India a testimonial, expressive of the sentiments of regard and esteem which they entertain for his character. A preliminary meeting was held about three weeks ago at the Chambers of the Hon. Sir John Grant, when the subject was talked over, and it was resolved that a book should be circulated among Dr. Martin's patients now resident in Calcutta, for the purpose of affording them the opportunity of subscribing to the proposed testimonial. Nearly one hundred gentlemen have entered their names; and a meeting was held yesterday afternoon at the Town Hall, in order to consider and decide in what manner the sum subscribed, which is very handsome, should be appropriated so as best to accomplish the object of the subscribers. Sir John Grant was called to the Chair, and in his usual succinct and appropriate style explained the purpose of the meeting. The following resolutions were then proposed and unanimously sustained:—

1. Moved by J. F. Leith, Esq. and seconded by Colonel Taylor, that the amount of the Subscriptions be remitted to Messrs. Rundle and Bridge of London, subject to the order of Sir Charles D'Oyley, Bart., James Young, Esq. and J. A. Dorin, Esq. and any two of them, and that these gentlemen or any two of them be requested to put themselves in communication with Dr. Martin, and to order from the said Messrs. Rundle and Bridge, such a piece or service of plate as they or any two of them may determine upon in communication with Dr. M. to the value of the amount remitted.

2. Moved by J. Allan, Esq. and seconded by G. Henderson, Esq. that the money be forthwith collected on Mr. Leith's receipt as Honorary Secretary, and that the amount be invested in the purchase of Treasury Bill or Bills payable to Messrs. Rundle and Bridge.

3. Moved by the Rev. W. H. Meiklejohn and seconded by C. W. Smith, Esq., that a letter be presented to Dr. Martin expressive of the regard and esteem of his patients, to be signed by the Hon. Sir John Peter Grant as chairman of this meeting, and to have the names of the subscribers to the testimonial appended to it; and that the Hon. Sir J. P. Grant be requested to write the letter.

These resolutions having been carried unanimously, it was agreed that a deputation wait on Dr. Martin, to present the letter, and that it be composed of the following gentlemen:—

The Honorable Sir J. P. GRANT,
Colonel TAYLOR,
The Rev. Dr. CHARLES,
C. W. SMITH, Esq.
J. ALLAN, Esq. and
J. F. LEITH, Esq. *Hon. Secretary,*

and that the Secretary do write to Dr. M. to request him to fix an hour on Saturday next for receiving the deputation.

Thanks were then voted to Sir J. P. Grant for his kindness in presiding and for his conduct in the chair, and the meeting separated.

We beg to tender our cordial congratulations to Dr. Martin on his receiving such a flattering testimony of regard for his personal character, and esteem for his professional services. We should have been sorry, indeed, if a gentleman, who is so exemplary in all the relations of life, and who has long stood in the first rank of his profession, had been permitted to leave the scene of his labours, without having been furnished with some mark of the esteem in which he is held. It is not in India as elsewhere. From the perpetually shifting character of the European portion of the population, all that any man, however distinguished in his profession, can hope to enjoy is contemporaneous reputation; for a more lasting fame falls to the lot of those alone—how few they are!—whose names are linked to some valuable improvement in the financial administration of the country, or who have covered themselves with glory in the battle-field, and by their martial achievements have added to the territorial possessions of the British Crown. Dr. Martin has, in his own walk, earned for himself a reputation, of which any one might be proud. His sterling integrity of character, his gentlemanlike propriety of conduct, his careful avoidance of professional bickerings, and his zealous devotion to the duties of his profession have been fully appreciated by his fellow-citizens; and we rejoice that he is to carry with him such a substantial token of the sentiments with which he is regarded by them. We sincerely hope that his projected voyage to Europe will contribute to the restoration of his health, and he has our best wishes that, in the future scene of his professional exertions, he may meet with the success of which he is so eminently deserving."

### TESTIMONIAL.

Dear Sir, *Calcutta, Jan.* 18*th*, 1840.

We, whose names are annexed to this letter, your friends and patients, while expressing our regret at losing the pleasure of your society and the benefits of your medical skill, and our still greater regret at your return to Great Britain being rendered necessary by the state of health, produced by your arduous services and your unremitting devotion to the duties of your profession, desire to present you with a lasting though inadequate memorial of our sincere regard for the excellencies of your personal character—our high estimate of your professional ability, and our gratitude for the benefit derived from its prompt, careful, and well-adapted exertion in the cases of ourselves and our friends.

We have for this purpose requested Sir Charles D'Oyly, Bart., James Young, Esq., and John Alexander Dorin, Esq., or any two of them who shall happen to be in London at the time of your arrival, to order a piece or pieces of plate to be made by Messrs. Rundell, Bridge, and Co. of London, of such description as you shall do us the favor to fix on, of the value of four hundred guineas, with a suitable inscription, of which we request your acceptance.

Signed by the Hon. Sir John Peter Grant, chairman of the meeting of the friends and patients of J. R. Martin, Esq. held at the Town Hall of Calcutta, in January, 1840.

Signed in their name and by their appointment.

JOHN PETER GRANT.

Then follow upwards of a hundred names of gentlemen of Calcutta.

